# Pressure Clamping During Ocular Perfusions Drives Nitric Oxide-Mediated Washout

**DOI:** 10.1167/iovs.64.7.36

**Published:** 2023-06-26

**Authors:** Ruth A. Kelly, Fiona S. McDonnell, Michael L. De Ieso, Darryl R. Overby, W. Daniel Stamer

**Affiliations:** 1 Ophthalmology Department, Duke University, Durham, North Carolina, United States; 2 Ophthalmology Department, University of Utah, Utah, United States; 3 Department of Bioengineering, Imperial College London, London, United Kingdom

**Keywords:** ocular perfusion, porcine, pressure clamping, washout, nitric oxide, outflow facility, trabecular meshwork

## Abstract

**Purpose:**

The aim of this study was to test the hypothesis that nitric oxide (NO) mediates a pressure-dependent, negative feedback loop that maintains conventional outflow homeostasis and thus IOP. If true, holding pressure during ocular perfusions will result in uncontrolled production of NO, hyper-relaxation of the trabecular meshwork, and washout.

**Methods:**

Paired porcine eyes were perfused at constant pressure of 15 mm Hg. After 1 hour acclimatization, one eye was exchanged with N5-[imino(nitroamino)methyl]-L-ornithine, methyl ester, monohydrochloride (L-NAME) (50 µm) and the contralateral eye with DBG, and perfused for 3 hours. In a separate group, one eye was exchanged with DETA-NO (100 nM) and the other with DBG and perfused for 30 minutes. Changes in conventional outflow tissue function and morphology were monitored.

**Results:**

Control eyes exhibited a washout rate of 15% (*P* = 0.0026), whereas eyes perfused with L-NAME showed a 10% decrease in outflow facility from baseline over 3 hours (*P* < 0.01); with nitrite levels in effluent positively correlating with time and facility. Compared with L-NAME–treated eyes, significant morphological changes in control eyes included increased distal vessel size, number of giant vacuoles, and juxtacanalicular tissue separation from the angular aqueous plexi (*P* < 0.05). For 30-minute perfusions, control eyes showed a washout rate of 11% (*P* = 0.075), whereas DETA-NO–treated eyes showed an increased washout rate of 33% from baseline (*P* < 0.005). Compared with control eyes, significant morphological changes in DETA-NO–treated eyes also included increased distal vessel size, number of giant vacuoles and juxtacanalicular tissue separation (*P* < 0.05).

**Conclusions:**

Uncontrolled NO production is responsible for washout during perfusions of nonhuman eyes where pressure is clamped.

For nearly 70 years, the washout effect has been an unexplained phenomenon that occurs during ocular perfusion of most species, except for humans and mice.^1^ The majority of these perfusions use the constant pressure perfusion technique.^2^^–^^5^ The effects of washout have also been observed during constant flow perfusions using porcine organ culture system^6^ and using a two-level constant pressure method with monkey organ-culture system.^7^ This phenomenon is defined as the decrease in resistance to outflow per volume of perfusate, even if aqueous humor (AH) is in the perfusate.^3^^,^^4^^,^^8^ In other words, washout occurs as a steady increase in outflow facility throughout ocular perfusion. Originally, washout was thought to be a time-dependent increase in outflow^4^; however, other studies observed that washout is a volume-dependent increase in outflow facility.^9^ The term washout was coined because early investigators hypothesized that extracellular glycosaminoglycan material was washing out of the outflow pathway.^5^^,^^10^^–^^12^ However, subsequent studies observed no change in either hyaluronic acid or proteoglycan content in the outflow pathway after perfusion.^13^^,^^14^ Interestingly, washout does not occur in human or mouse eyes,^1^^,^^3^^,^^15^ suggesting unique anatomical or physiological properties in the outflow pathway of both these species.^1^^,^^15^ In mice, as in humans, the cribriform elastin network is very complex^16^^,^^17^ and is likely holding together the juxtacanalicular tissue (JCT), preventing washout. The current thinking is that the lack of washout, observed in human and mouse eyes, is due to this elaborate connectivity between the ciliary muscle and the inner wall (IW) of Schlemm's canal (SC) and the JCT region. The cribriform elastin network provides this support system, which is less complex in nonhuman primates and other nonhuman species.^15^ It is unclear why nonhuman primate eyes, which are anatomically similar to human eyes, exhibit severe washout.^4^^,^^11^

The JCT in all species is critical because the bulk of AH outflow resistance is located in this region of the conventional outflow pathway.^18^^–^^21^ So, understanding the mechanisms that underlie the washout effect in nonhuman eyes may inform the unique physiology of human eyes and/or important homeostatic regulators of conventional outflow.^22^ For example, when pressure is clamped during ocular perfusion experiments, the JCT expands and a physical separation between the IW and JCT occurs. This separation eliminates the downstream boundary condition that restricts flow through the JCT and seems to short circuit the funneling of AH, likely contributing to washout.^23^^,^^24^ Thus, an expanded JCT and this IW–JCT separation allows the AH to have greater access to the IW, instead of being funneled to confined regions of the IW that amplifies resistance.^25^ In humans, the JCT resists such expansion and separation from the IW, likely owing to its elaborate cribriform elastin network.^18^

Previous studies have shown that the effects of washout in bovine eyes are reversible and correlate with IW/JCT separation, which can take two distinct forms, namely, cell–matrix separation between the IW and basal lamina and matrix–matrix separation between the basal lamina and ECM within the JCT itself.^15^^,^^23^^,^^24^ IW–JCT separation was identified using light and electron microscopy and based on expansion of the JCT and ballooning of the IW protruding into the lumen of the AAP, as described by Lu et al.^24^ Washout, JCT expansion, and thus increased outflow facility can be imitated in bovine eyes by rho-associated kinase (ROCK) inhibitors,^24^^,^^26^ which cause trabecular meshwork (TM) cell relaxation in vitro and ex vivo.^27^ In fact, most TM-relaxing drugs decrease outflow resistance. In contrast, most agents that contract the TM decrease outflow facility, emphasizing the important role of TM contractility and, thus, flow dimensions in JCT on outflow resistance.^27^^–^^34^ A notable exception is pilocarpine, which lowered the IOP and increased the outflow facility by preferentially contracting the ciliary muscle, preventing complete collapse of SC lumen under higher pressures in living mice.^35^

The vasodilator nitric oxide (NO) relaxes the TM and increases outflow facility.^36^^–^^38^ NO is produced in the conventional outflow pathway by SC endothelia and is regulated by shear stress in the SC lumen.^39^^,^^40^ The size of the SC lumen and, thus, shear stress is determined by the pressure gradient across the TM, which is determined by the difference between the episcleral venous pressure and the IOP,^40^^–^^42^ and the flow rate through the conventional outflow pathway. Therefore, IOP-mediated increases in NO released by SC cells likely regulates the contractility of the underlying TM.^42^ Significantly, these relationships establish a homeostatic mechanism that regulates resistance to AH outflow and, therefore, IOP.^38^^,^^42^^,^^43^ In support of an essential role for NO in IOP homeostasis, transgenic mice overexpressing endothelial NO synthase (eNOS) have lower IOPs and increased outflow facility, whereas eNOS knockout mice have higher IOPs and decreased outflow facility.^44^^,^^45^ Importantly, polymorphisms in the NOS3 gene in humans, which encodes eNOS, has been associated with an increased risk of glaucoma.^46^

Taken together, the present study tests the premise that NO mediates a pressure-dependent, negative feedback loop maintaining conventional outflow homeostasis and thus IOP. Specifically, we hypothesize that, when pressure is clamped, NO acts to decrease resistance, which increases flow. At the same time, the pressure drop remains clamped and relaxation of the TM by NO exacerbates collapse of the SC. Both effects increase shear stress in the SC, and hence further increase NO in a feed-forward manner, which destabilizes the system. To test this hypothesis, we attempt to prevent washout in eyes under constant pressure with N5-[imino(nitroamino)methyl]-L-ornithine, methyl ester, monohydrochloride (L-NAME), a NO synthase inhibitor, in the perfusion media and mimic washout by short-term perfusion with a NO donor (DETA-NO).

## Materials and Methods

### Porcine Eyes

Porcine eyes were obtained from a local abattoir (Neese Sausage, Burlington, NC) and were kept on ice until start of perfusion (6–8 hours post mortem). Each eye used in the study was confirmed to be grossly normal by examination under a dissecting microscope, and eyes with any noticeable damage during processing at abattoir were not perfused.

### Whole Globe Perfusions

#### Perfusion Media

All eyes were perfused at 37°C with Dulbecco's phosphate-buffered saline (Life Technologies, Grand Island, NY) containing 5.5 mM glucose, referred to as DBG. L-NAME was obtained from Sigma-Aldrich (St Louis, MO). DETA-NONOate (DETA-NO) was obtained from Cayman Chemical Company (Ann Arbor, MI) (Batch #:0633762-1). A new vial of L-NAME or DETA-NO was opened on the day of each perfusion, owing to their short shelf life. DETA-NO was protected from light at all times as per manufacturer's recommendations. L-NAME and DETA-NO were diluted in DBG, with final working concentrations of 50 µM and 100 nM, respectively.

#### Perfusion Routine

Paired porcine eyes were used in all perfusions. A modified version of the iPerfusion system was used for whole eye, porcine perfusions (Supplementary Fig. 1A). The iPerfusion system was developed to measure conventional outflow facility in mouse eyes, and our laboratory adapted it for porcine eyes. In this modified version, reservoirs delivering drug or vehicle and DBG were set at a height equal to 15 mm Hg. Tubing and stopcocks were connected to reservoirs and flow sensor, which monitors flow throughout the perfusion. The flow and pressure sensors on the manifold are connected to the primary reservoir at a height of 15 mm Hg. Tubing from this was connected to a 25G needle, which was cannulated through the cornea and pupil, positioned with the bevel of the needle facing upwards in the eye's posterior chamber (to deliver perfusion media and prevent anterior chamber deepening, observed during ex vivo mouse perfusion when the needle is placed in the anterior chamber^1^^,^^47^). A second needle was inserted into the anterior chamber, bevel up, and connected to a waste or exchange reservoir that was closed off during the perfusion and opened during anterior chamber exchanges (Supplementary Fig. 1B). Before cannulations, all eyes were cleaned of extraocular tissue and submerged to the limbus in a water bath containing DBG. A moist piece of gauze was draped over the eye to keep the eye from drying out throughout the perfusion. If there was an air bubble or leak that occurred during the course of the perfusion, the pair was excluded from analysis. Treated eye (OD vs OS) was alternated between experiments and not chosen based on starting facility values.

To begin each experiment, eyes were perfused with DBG for 1 hour for acclimatization and to establish a stable baseline outflow facility. Anterior chamber exchange using a pressure difference of 5 mm Hg between the primary reservoir and the waste reservoir (5 mL exchange volume over approximately 20 minutes) was carried out with either DBG containing 50 µM L-NAME or DBG alone (*n* = 7 pairs total) for L-NAME experiments, and DBG containing 100 nM DETA-NO or DBG alone (*n* = 6 pairs total) for DETA-NO experiments. A drop of India Ink was added to the perfusate in order to visualize the exchange of fluid from the 15 mm Hg reservoir into the waste reservoir. The waste reservoir was then closed off and the eye was once again perfused under a constant pressure of 15 mm Hg and perfusions were run for 3 hours for L-NAME experiment and 30 minutes (short-term perfusion) for DETA-NO experiment (Supplementary Fig. 4). Previous studies have shown that 3 hours of perfusion at 15 mm Hg is sufficient to observe washout in bovine and porcine eyes.^2^^,^^15^^,^^22^^–^^24^ To label the hydrodynamic patterns of the outflow, eyes were exchanged with 5 mL of DBG containing yellow/green fluorescent microspheres (FluoSphere, 0.2 µm diameter Lot #: 2268375; ThermoFisher Scientific, Waltham, MA) at 0.02% v/v followed by a 30-minute perfusion. Eyes were then perfusion fixed at 15 mm Hg, beginning with an exchange of 5 mL of Karnovsky's fixative (2.5% glutaraldehyde and 2% paraformaldehyde in PBS), followed by 30 minutes of perfusion. Globes were cut along the equator and eyes were left in Karnovsky's fixative solution over night at 4°C. Fixative was exchanged for PBS and eyes were kept in the dark at 4°C.

### iPerfusion Data Analysis

Customized iPerfusion software recorded raw facility values throughout the perfusion (multifunction DAQ, National Instruments Corp, Austin, TX).^48^ These raw facility values were then compressed using DownSamplePost. MATLAB was then used to extrapolate facility vs time. These data were copied into Excel and the average facility values for the acclimation step and specific time points (at 30 minutes, 1 hour, 2 hours, and 3 hours) throughout the perfusion were calculated. For each step, approximately 150 to 200 readings were taken and averaged over 30 minutes for each of the 30-minute, 1-hour, 2-hour, and 3-hour time points. Overall outflow facility (%) was calculated as the change in facility at 3 hours (L-NAME) or 30 minutes (DETA-NO), compared with the acclimation facility value. The results were displayed using GraphPad Prism 9.4.1 (GraphPad, La Jolla, CA).

### Nitrite Assay

Whole eyes were placed in specially designed plastic baskets positioned above a funnel-shaped custom perfusion chamber for efficient sampling of effluent produced during the course of the perfusion. Eyes and chambers were placed in a water bath to control humidity and temperature. NO concentration was indirectly assessed by measuring the concentration of nitrite, a by-product of NO degradation, with a Measure-IT High-Sensitivity Nitrite Assay Kit according to the manufacturer's directions (Invitrogen by Life Technologies, Grand Island, NY). Effluent was collected at 1 hour, 2 hours, and 3 hours of the L-NAME versus DBG control perfusion and stored at –80°C. All of the effluent was collected from the bottom of the funnel shaped chamber at each time point. From each effluent sample, 100 µL of each was concentrated 4× using a speed vacuum system. Concentration of effluent samples was necessary so that nitrite levels fell in the linear range of the standard curve. Samples were then loaded into a 96-well plate (10 µL/well), combined with a working solution and developer, as per the manufacturer's protocol. The fluorescence of the nitrite-containing samples was measured at using a SpectraMax M5 plate reader coupled with SoftMax Pro 7 software (Molecular Devices, Sunnyvale, CA). Briefly, a standard curve was made using serial dilutions of a nitrite standard included in the kit. Linear regression analysis using data from the standard curve was used to estimate the nitrite concentrations of the samples. Nitrite concentration (nanomoles) was calculated for each effluent sample taken at an hourly interval, taking into account the 4× concentration carried out using the speed vacuum system (Supplementary Table 1). The rate of nitrite production (picomoles per hour) at each of these hourly intervals was then calculated based on the volume of effluent collected at each time point.

### Light Microscopy

Eyes were hemisected along the equator, the lens and vitreous removed and anterior segment were cut into four quadrants. For gross morphology studies, two samples (approximately 1.0–1.5 mm thick) were cut from four quadrants of each eye (*n* = 8 total samples per eye). Samples were selected only from flow conducting regions (medium to high flow), as indicated by labeling from the fluorescent tracer (Supplementary Fig. 6). Each sample was embedded in Epon and 0.5 µm semithin sections were cut, stained with 1% of methylene blue and examined by light microscopy (Axioplan2, Carl Zeiss MicroImaging, Thornwood, NY).

### Morphological Analyses

Visualized by light microscopy in semithin sections, we defined IW–JCT separation as the separation of the IW of the angular aqueous plexus (AAPs) from the underlying JCT region (often ballooning into the lumen), which is consistent with previous definitions.^15^^,^^24^ To quantitatively examine changes in the IW AAP/JCT separation and size of AAP/distal vessels (DVs) two trained masked observers measured the length of the IW of AAP, circumferential length of AAPs and DVs, number of giant vacuoles (GVs) and percentage separation length (PSL) in deidentified images in all treatment and control groups. Masked observers carried out morphological measurements on coded images, displaying the results in Excel. After this, the Excel file was decoded and results displayed and statistical analysis carried out by the lead scientist using GraphPad Prism 9.4.1. Mean values for each measurement ± SD from the mean is displayed for both the L-NAME and DETA-NO groups.

#### Length of AAP

The length of each AAP was measured using the straight-line tool on ImageJ, measuring from the anterior to poster extremity of the IW of AAP. If an image had more than one AAP, the length of each AAP was measured and the average of all AAPs for that section was calculated. The length of the AAPs in each quadrant was then combined to derive the average for each eye.

#### Circumferential Length of AAP and DVs

The circumference of each AAP and DV were measured using the freehand line tool on ImageJ, whereby the circumference of the lumen was carefully drawn for each AAP and DV. The circumference of each AAP was averaged for each sample. The circumference of each quadrant was also averaged to obtain the overall average for each eye. DVs were identified based on their location in the intra to episcleral region, their distinctive circular shape and tunics, or different tissue layers within its lumen. The morphology of DVs were examined which included episcleral veins, but excluded episcleral arteries. Episcleral arteries, found in less abundance, were identified by their typical circular appearance enclosed by muscular walls, as previously described by Ren at al.^49^ Episcleral veins measured will be referred to as DVs throughout the text.

#### GV Counting

The average number of GVs in each AAP were counted manually for each quadrant and averaged for each eye. A GV was identified as a large unshaded outpouching of the IW of AAP into its lumen.

#### PSL

The total length of each AAP was calculated as described above as well as the length of the IW of AAP that was separated from the JCT region, based upon IW separation or ballooning of the IW that protrudes into the lumen of the AAP (Supplementary Fig. 5), as described by Lu et al.^24^ The separated length of the AAP was divided by the total length of the AAP and then multiplied by 100 to give the PSL.^24^^,^^50^ Therefore, if there was no separation, the PSL = 0% and if all of the AAP showed IW/JCT separation, the PSL = 100%. If an image had more than one AAP, the average of all AAP measurements were taken for that image. The PSL of each image was calculated and, for each quadrant and the average of this, was calculated for each eye.

### Statistical Analysis

Paired student *t*-tests, one-way ANOVA with Tukey's multiple comparisons test, and one-sample *t*-test to a theoretical mean of 0 were used using GraphPad Prism 9.4.1 for statistical analysis. ANCOVA analysis was used to measure difference in nitrite vs facility in DBG control and L-NAME eyes, separately. Data were considered significantly different if *P* < 0.05.

## Results

### Whole Globe Perfusions With L-NAME

**Figure 1.**
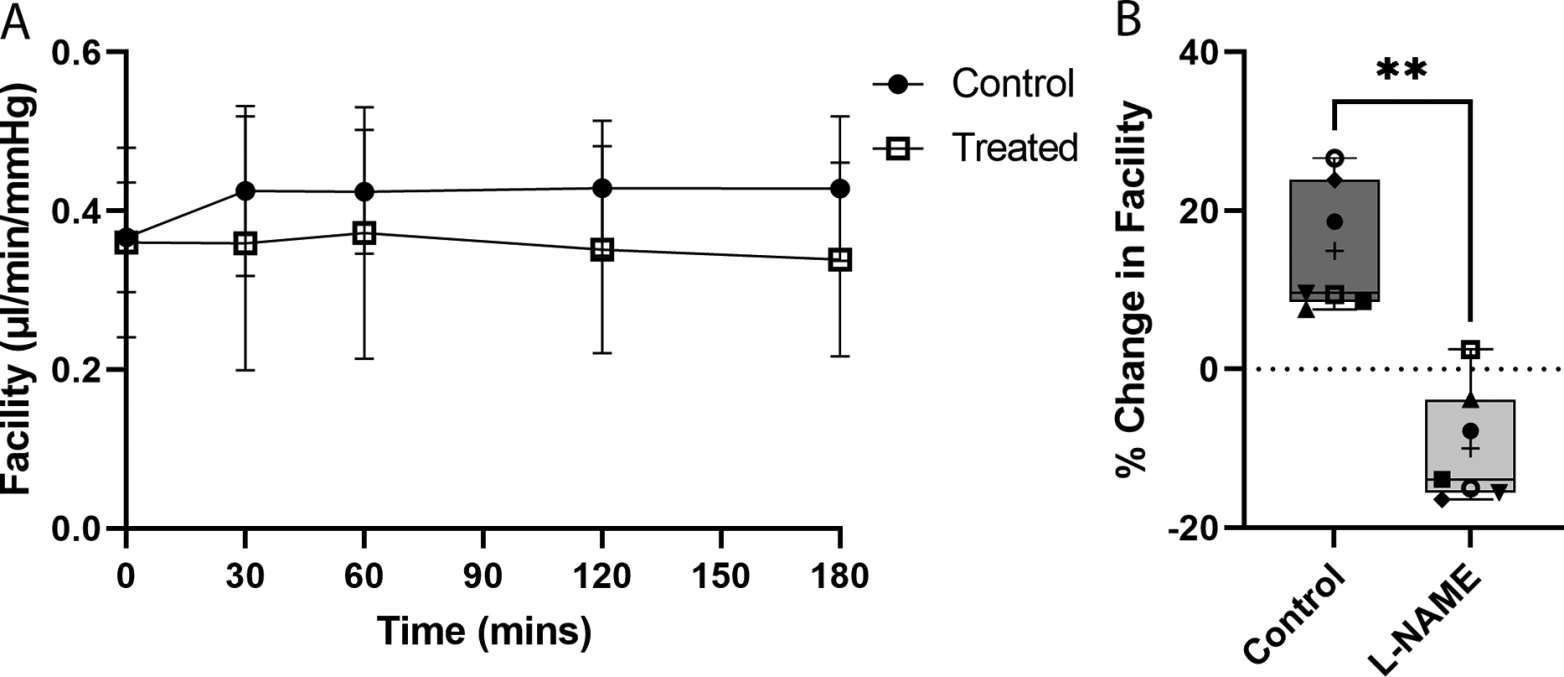
L-NAME prevents washout in porcine eyes perfused at constant pressure. (**A**) Facility values for all pairs of eyes perfused at constant pressure for 3 hours. Control eyes exhibited washout (increase in outflow facility) over the 3 hours. In contrast, L-NAME (50 µM)–perfused eyes showed a steady decrease in outflow facility after 60 minutes of treatment with L-NAME. Data points represent mean facility values with error bars showing ± SD from the mean. (**B**) Paired analysis shows control eyes exhibited a 15% increase in outflow facility from baseline (*P* = 0.003). In contrast, eyes perfused with L-NAME (50 µM) showed a steady decrease in outflow facility of 10% from baseline (*P* = 0.01), with a net decrease of 25% compared with DBG control eyes (*P* = 0.002). Box and whisker plots display minimum to maximum, with the mean displayed as + and horizontal line in box indicates median on each graph at end of perfusion. Dotted line indicates starting outflow facility. Statistical analysis was carried out using one sample *t*-test to theoretical mean of 0, indicating no change from baseline and paired *t*-test, and *n* = 7 pairs. Each individual data point corresponds with each pair. **P < 0.01.

To examine the role of NO synthase in washout, one eye of porcine pairs was perfused with L-NAME (50 µM) in DBG, while the contralateral eyes were perfused with DBG (Fig. 1A). Baseline facility values for control and L-NAME–treated eyes were 0.37 ± 0.1 and 0.36 ± 0.1 µl/min/mm Hg respectively (*P* = 0.9034, *t*-test, *n* = 7) (Supplementary Fig. 2A). Control eyes perfused with DBG demonstrated washout over the course of 3 hours of perfusion, with an overall increase in outflow facility of 14.9 ± 8.0% from baseline (mean ± SD, *n* = 7, *P* = 0.003). In contrast, L-NAME–treated eyes showed a steady *decrease* in outflow facility over the 3 hours, with a final decrease of 10.0 ± 7.2% from baseline (*P* = 0.01) and a net decrease of 25% compared with control eyes (*P* = 0.002, paired *t*-test, *n* = 7) (Fig. 1B). Thus, L-NAME–treated eyes not only eliminated washout, but appeared to further decrease outflow facility from baseline, presumably by inhibiting endogenous eNOS activity.

### Morphological Effects of L-NAME on Conventional Outflow Tissues

**Figure 2.**
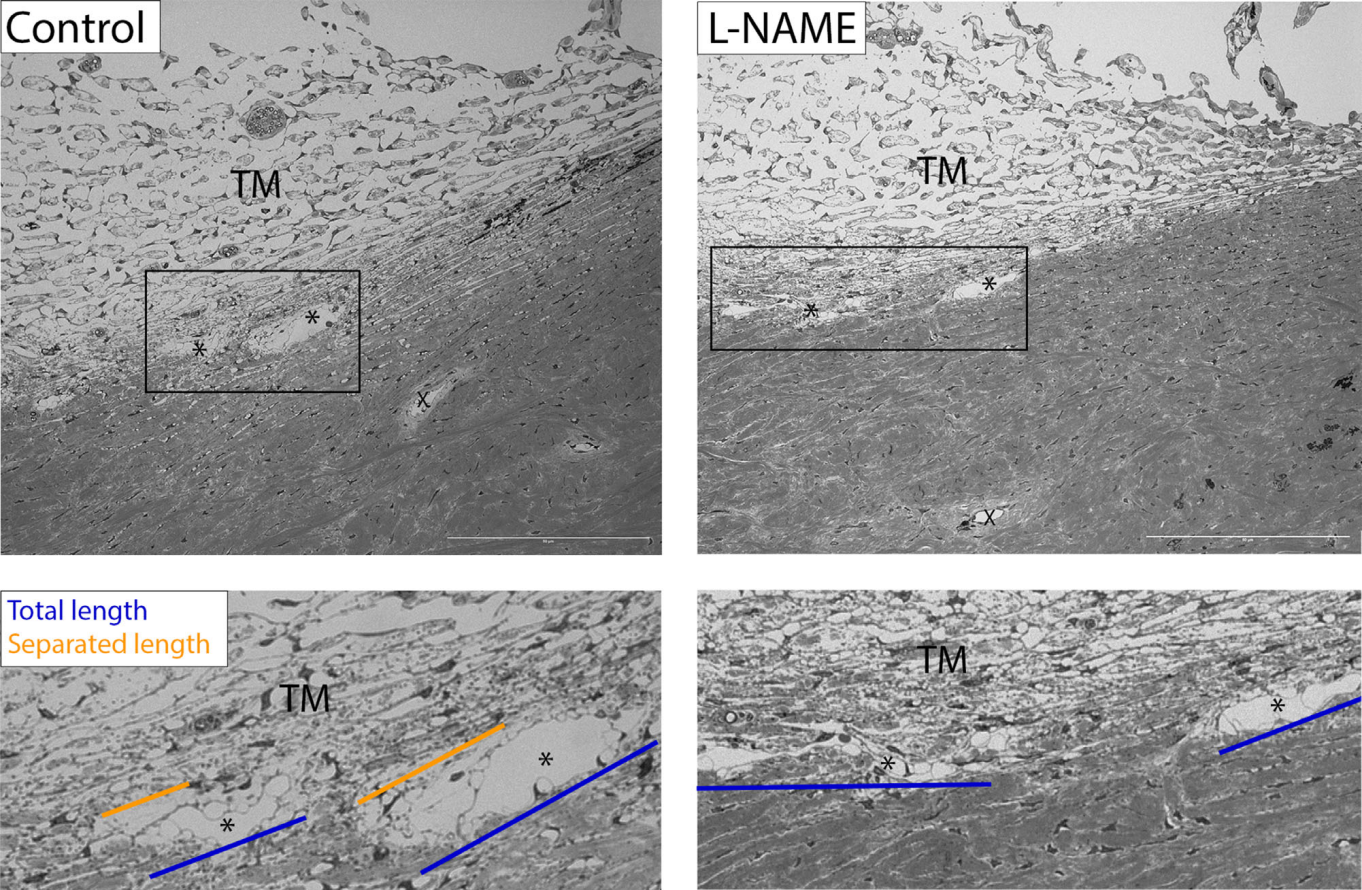
Morphology of conventional outflow tissues perfused with L-NAME versus control for 3 hours. Control eyes perfused with vehicle exhibited the effects of washout over the course of the 3-hour perfusion, showing increased expansion of the JCT region itself, as well as increased separation of the IW from the JCT region. Abundant GV formation was also apparent (*left*). In contrast, L-NAME–treated eyes showed no separation of the IW from JCT region. Narrowing of the AAP and surrounding tissue is obvious, compared with DBG control eyes, with fewer GVs present in the AAP (*right*). The total length of the AAP is shown in blue and separated length in orange. *AAP. X, DV.

Paired porcine eyes that were perfused for 3 hours with either L-NAME or DBG, were then perfused with tracer to label flow pathways. Eyes were analyzed for morphological changes of conventional outflow tissues having medium to high amounts of tracer labeling in sagittal sections, indicating active flow areas (Supplementary Fig. 6). Gross examination of control eyes shows the expanded JCT region and increased separated of the IW from the JCT region in the AAPs (Fig. 2, left), similar to that seen in previous studies of eyes exhibiting washout.^15^^,^^22^^,^^23^ Moreover, numerous GVs were apparent in AAPs of control eyes. However, in L-NAME–treated eyes the JCT region was more compact, with no separation of the IW from the JCT in the AAPs and GVs appeared qualitatively in fewer numbers (Fig. 2, right).

### Quantitative Analysis of Conventional Outflow Tissues in L-NAME–Perfused Eyes

**Figure 3.**
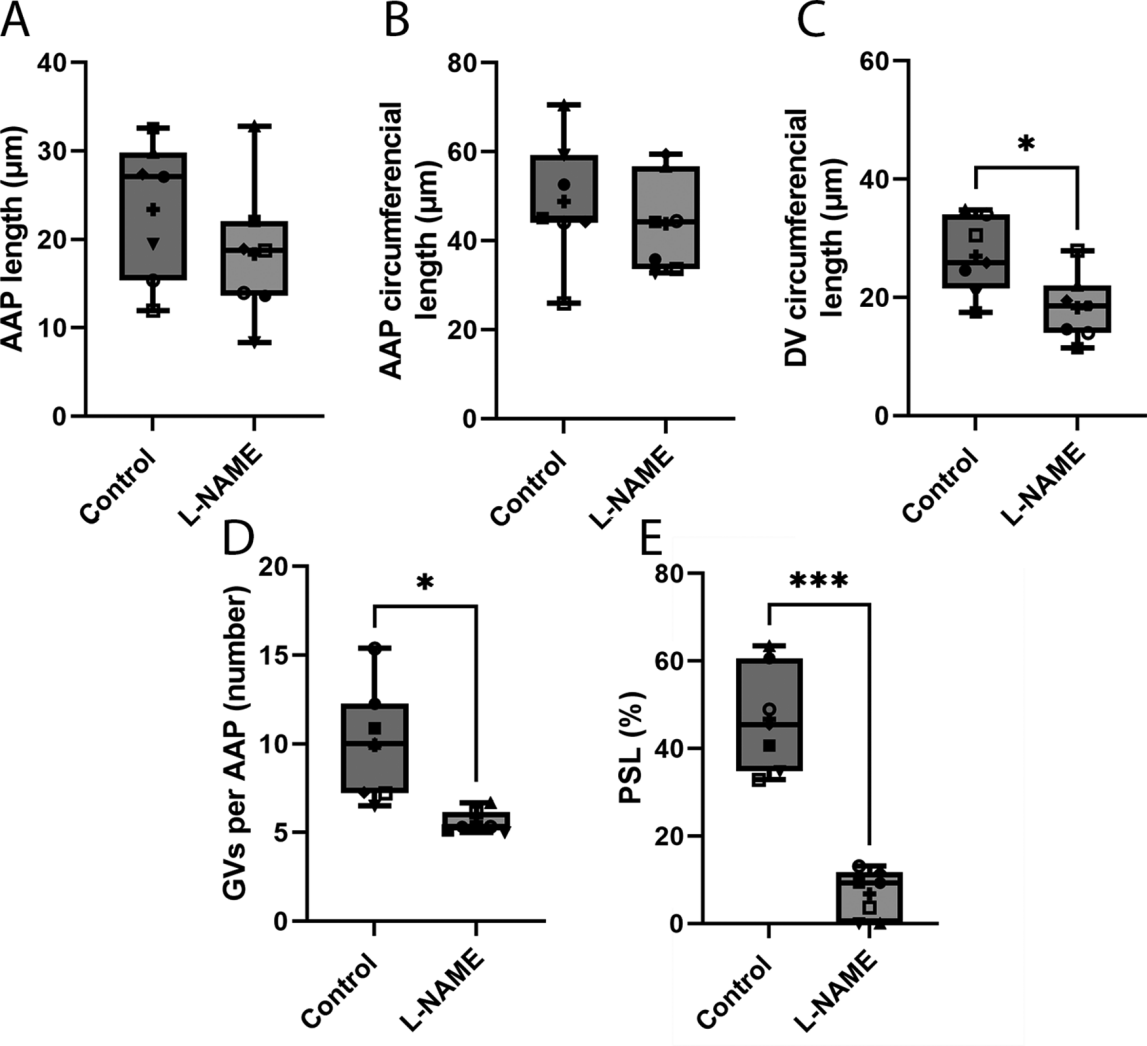
Quantitative analysis of conventional outflow tissue measurements in L-NAME versus control perfusions. There was no significant change in the AAP length (**A**) and circumferential length (**B**) of AAPs between control and L-NAME–treated eyes (*P* = 0.14 and *P* = 0.41, respectively). However, there was a significant decrease in the circumference of DVs in L-NAME–treated eyes compared with control eyes (*P* = 0.016) (**C**). There was also a significant reduction in the number of GVs present in each AAP in L-NAME–treated eyes compared with control eyes (*P* = 0.013) (**D**). Finally, there was a significant reduction in the PSL in L-NAME–treated eyes compared with control eyes (*P* = 0.0002) (**E**). Box and whisker plots display minimum to maximum with mean displayed as + and horizontal line in box showing median on each graph. Each individual data point corresponds to each pair. Statistical analysis was carried out using paired *t*-test, *n* = 7 pairs. *P < 0.05, ***P < 0.001.

Measurements were carried out to quantify length and circumference of AAPs, circumference of DVs, the number of GVs per AAP and the PSL of each AAP. Results are displayed as the mean ± SD for each measurement. Results show no significant difference in the length and circumference of AAPs (*P* = 0.14 and *P* = 0.41, respectively *n* = 7) in control eyes exhibiting washout compared with L-NAME–treated eyes (Fig. 3A+B). Mean AAP length and circumferential length in DBG washout eyes was 48.8 ± 14.0 µm and 23.4 ± 7.8 µm respectively, compared with 43.8 ± 10.8 µm and 18.4 ± 7.8 µm respectively in L-NAME–treated eyes.

In contrast, the mean DV circumferential length in L-NAME–treated eyes seemed to be significantly decreased compared with control eyes (from 27.0 ± 6.5 µm to 18.3 ± 5.6 µm; *P* = 0.01; *n* = 7) (Fig. 3C). Moreover, the number of GVs per AAP in L-NAME–treated eyes were significantly decreased in number compared with control (9.9 ± 3.2 vs. 5.6 ± 0.6 GVs per AAP; *P* = 0.01; *n* = 7) (Fig. 3D). Finally, the PSL (PSL = separated length of AAP/total length of AAP × 100) in L-NAME–treated eyes was significantly decreased compared with control (from 46.7 ± 11.9% to 6.7 ± 5.5%; *P* = 0.0002; *n* = 7; paired *t*-test) (Fig. 3E).

### Nitrite Levels in DBG Control Eyes Compared With L-NAME–Treated Eyes

**Figure 4.**
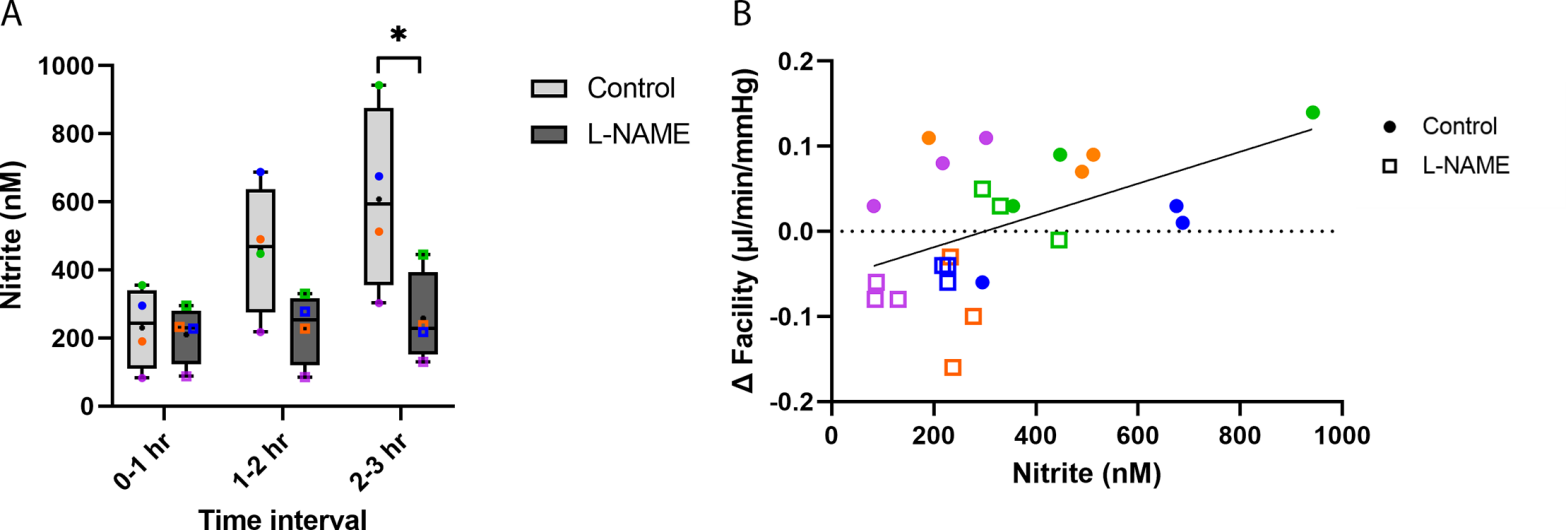
Nitrite levels in effluent from perfused eyes increases with time in eyes demonstrating “washout” and positively correlates with outflow facility. In a separate group of porcine whole globes perfused at constant pressure of 15 mm Hg (*n* = 4), effluent was collected once every hour during the perfusion and analyzed for nitrite content to estimate NO concentration produced by conventional outflow cells. (**A**) Increased nitrite levels were observed over time in DBG control eyes compared with L-NAME–treated eyes, which was statistically significant at 2- to 3-hour intervals of perfusion (*P* = 0.0196). (**B**) There was a positive relationship between facility change (Δ Facility) and nitrite levels, indicating that increased facility is associated with an increase in nitrite concentration (R^2^ = 0.2544). Individual data points are displayed above and each pair of eyes is associated with a particular color.

In a separate cohort of paired eyes, the anterior chamber of one eye was exchanged with L-NAME (50 µM) after 1 hour of acclimation, while the controlateral eye recived DBG and was perfused at constant pressure of 15 mm Hg for 3 hours. As before, DBG control eyes exhibited washout (19 ± 9.3% increase in outflow facility over 3 hours) compared with L-NAME–treated eyes (14 ± 9.4% decrease in outflow facility over 3 hours) (Supplementary Table 1). Effluent from both eyes was collected once per hour over the course of 3-hour perfusions to asses NO concentration produced by conventional outflow cells. Analysis of covariance was used to examine treatment groups individually. A statistically significant increase in nitrite over time (*P* = 0.0032) was observed in DBG eyes, with a difference in nitrite levels observed between pairs (*P* = 0.02). In L-NAME–treated eyes, there was no statistically significant relationship between nitrite and time (*P* = 0.06). There was a difference observed between pairs, however (*P* = 0.0013). This analysis supports the hypothesis that nitrite increases with time in control eyes, but not in L-NAME–treated eyes. This statistical analysis does not account for paired interactions between groups and so nitrite (nanomoles) was plotted versus the hourly time intervals when effluent was taken (Fig. 4A). In effluent, we observed increased nitrite levels over time in DBG control eyes compared with L-NAME–treated eyes. The mean nitrite levels of 230.6 ± 103.9 nM, 460.6 ± 167.0 nM, and 608.1 ± 233.9 nM were calculated from effluent collected at 0- to 1-hour, 1- to 2-hour, and 2- to 3-hour intervals of perfusion in control eyes, respectively. In contrast, mean nitrite levels of 210.625 ± 75.9 nM, 230.0 ± 91.2 nM, and 257.5 ± 115.6 nM were observed in L-NAME–treated eyes at the same time points. A significant difference in nitrite concentration was observed between control and L-NAME–treated eyes at 2- to 3-hour intervals of perfusion (*P* = 0.0196; paired *t*-test; *n* = 4) (Fig. 4A). When taking into account effluent volume, nitrite production was significantly elevated in DBG control eyes compared with L-NAME–treated eyes over time at the 1- to 2-hour (*P* = 0.0114; paired *t*-test; *n* = 4) (Supplementary Fig. 3) and 2- to 3-hour (*P* = 0.0406) intervals. Nitrite production was calculated based on the volume of effluent collected at each time point.

We next looked at the relationship between nitrite concentration and outflow facility. Analysis of covariance, using a separate line model, was used to account for variation in slopes between pairs. A positive relationship was observed between facility change and nitrite in control eyes (*P* = 0.011). However, this positive correlation between facility and nitrite observed in control eyes was eliminated in L-NAME–treated eyes (*P* = 0.38). Because facility and nitrite levels varied between pairs, we visualized the relationships by plotting nitrite (nanomoles) versus change in facility or Δ facility (microliters per minute per millimeter of mercury) for each interval where nitrite levels were measured (i.e., 0–1, 1–2, and 2–3 hours) (Fig. 4B). Change in facility was calculated as mean facility over the time interval minus baseline facility for each individual eye. The line of best fit through all data points (both control and L-NAME) shows an overall positive relationship between nitrite and facility, regardless of treatment (R^2^ = 0.2544).

### Whole Globe Perfusions With DETA-NO

**Figure 5.**
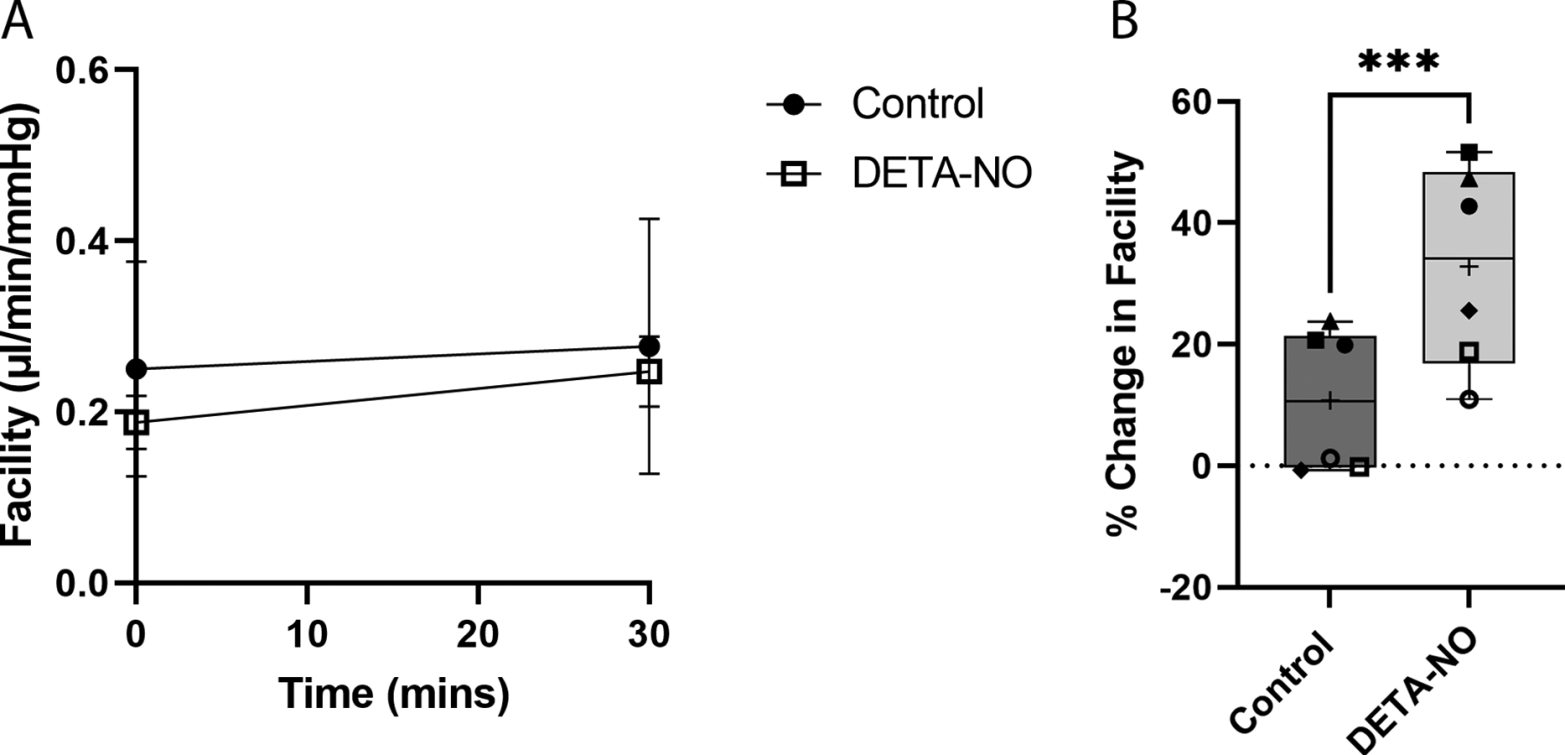
Short-term perfusion with DETA-NO accelerates washout in porcine eyes. (**A**) Facility values of all paired eyes perfused at constant pressure for 30 minutes. Control eyes showed a small increase in outflow facility over the 30-minute perfusion. DETA-NO (100 nM)–perfused eyes, however, showed an even greater increase in outflow facility. Data points represent mean facility values with error bars showing ± SD from the mean. (**B**) Paired analysis showed control eyes exhibiting a washout rate of 11% increase in outflow facility after 30 minutes of perfusion (*P* = 0.075). DETA-NO–treated eyes showed an increase in outflow facility of 33% after 30 minutes (*P* = 0.005), with a net increase of 22% compared with control eyes (*P* = 0.001, *n* = 6). Box and whisker plots display minimum to maximum values, with mean displayed as + on each graph. Statistical analysis was carried out using one sample *t*-test to theoretical mean of 0, indicating no change from baseline and paired *t*-test, and *n* = 6 pairs. Each individual data point corresponds to each pair. ***P < 0.001.

To test our hypothesis that chronic NO release drives washout, we perfused one eye of pair with 100 nM DETA-NO, while the contralateral eye was perfused with DBG for a short amount of time (30 minutes) (Fig. 5A). Baseline facility values for control and DETA-NO–treated eyes were 0.25 ± 0.1 and 0.19 ± 0.03 µL/min/mm Hg respectively (*P* = 0.2661; *t*-test; *n* = 6) (Supplementary Fig. 2B). Similar to our experiment with L-NAME, DBG control eyes showed a moderate but insignificant increase in outflow facility, or a washout rate of 10.8 ± 11.8% change from baseline (*P* = 0.075; *n* = 6) over this short time period. DETA-NO–treated eyes, however, showed an even greater increase in outflow facility of 32.8 ± 16.7% from baseline (*P* = 0.005) with a net increase of 22% compared with control eyes (*P* = 0.001, *n* = 6) over 30 minutes of perfusion (Fig. 5B).

### Morphology of DETA-NO Perfusions

**Figure 6.**
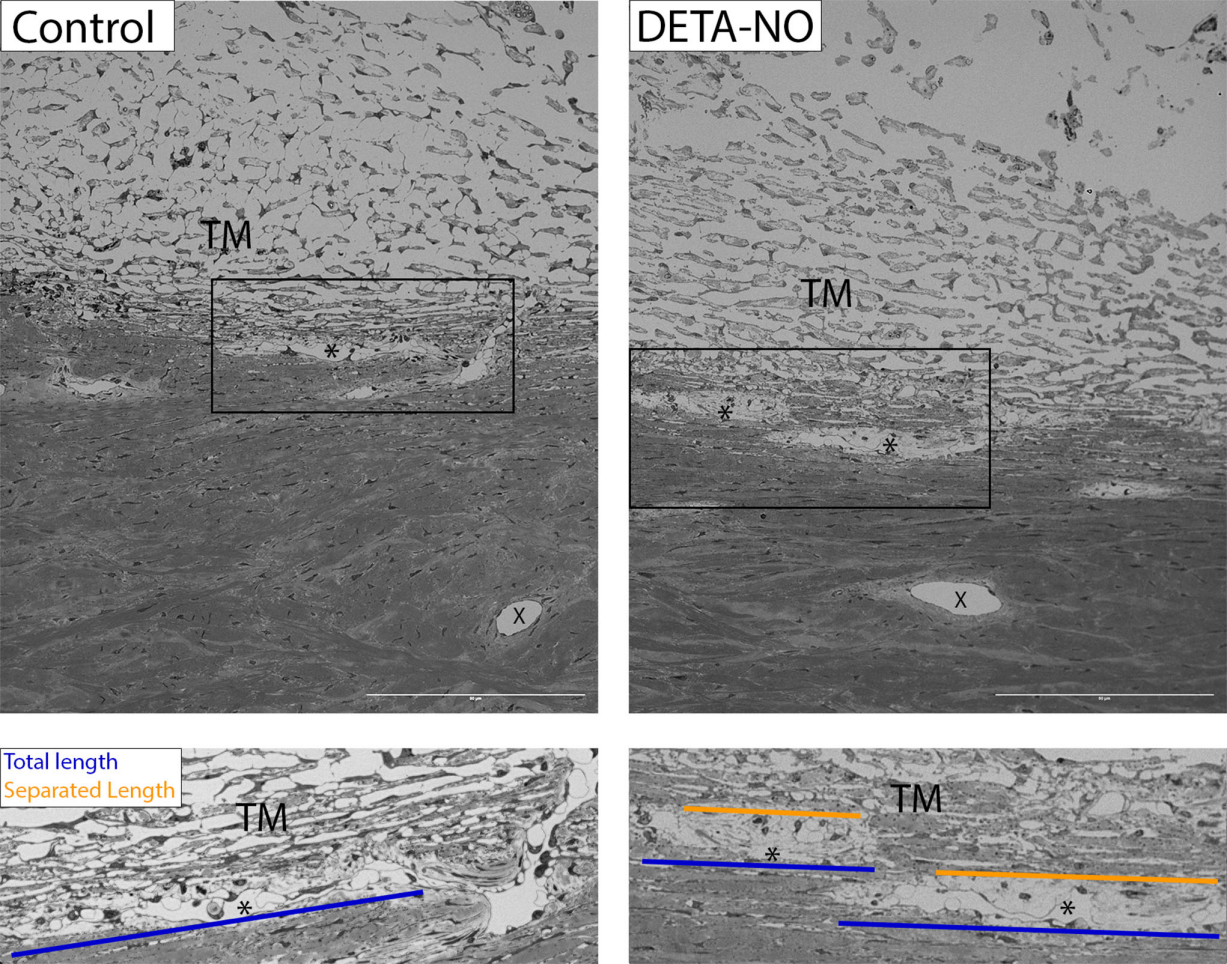
Morphology of conventional outflow tissues perfused for 30 minutes with DETA-NO versus control. After a 30-minute perfusion, the early effects of washout were not obvious in the control eyes. As such, there was very little expansion of the JCT and no separation of the JCT from the IW AAP in these eyes (*left*). The DETA-NO–treated eyes, however, displayed significant separation of the IW from the JCT, protruding into the AAP lumen, as well as expansion in the JCT region itself. There were abundantly more GVs in APPs, as well as AAP and DV dilation (*right*). Total AAP length is shown in blue and separated length in orange. *AAP. X, DV.

Morphological analysis shows little separation of the IW AAP from the JCT region in control eyes perfused for 30 minutes, with little evidence of changes consistent with (Fig. 6, left). In eyes perfused with DETA-NO for only 30 minutes, however, the separation of the IW AAP from the JCT is clearly apparent, as well as expansion of the JCT region (Fig. 6, right). The ballooning of the IW was exacerbated in the DETA-NO–treated eyes compared with the control eyes. There was an abundance of GVs present in the DETA-NO–treated eyes, compared with the controls. There seems to be a washout-like expansion in the JCT region in DETA-NO–treated eyes, with tissues seeming to be less tightly packed. Dilation of AAPs and DVs is obvious in the DETA-NO–treated eyes compared with the controls.

### Quantitative Analysis of DETA-NO Perfusions

**Figure 7.**
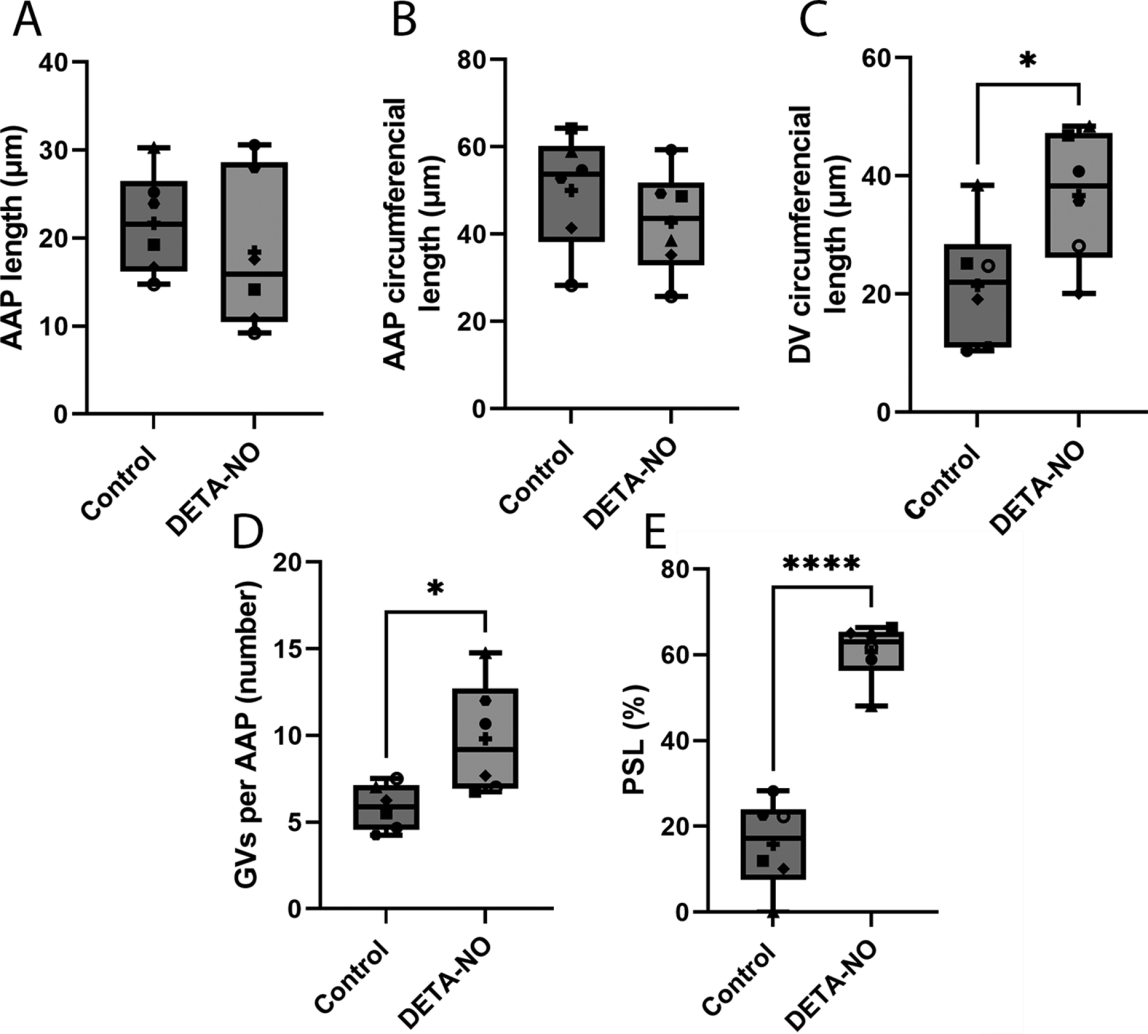
Quantitative analysis of conventional outflow tissue measurements in DETA-NO versus control perfusions. There was no significant change in the long axis (**A**) and circumferential length (**B**) of AAPs between control and DETA-NO–treated eyes (*P* = 0.42 and *P* = 0.11, respectively). However, there was a significant increase in the circumference of DVs in DETA-NO–treated eyes compared with control eyes (*P* = 0.03) (**C**). There was also a significant increase in the number of GVs present in each AAP in DETA-NO–treated eyes compared with control eyes (*P* = 0.046) (**D**). Finally, there was a significant increase in the PSL in DETA-NO–treated eyes compared with control eyes (*P* < 0.0001) (**E**). Box and whisker plots display minimum to maximum values with the mean displayed as + and horizontal line in box showing median on each graph. Each individual data point corresponds with each pair. Statistical analysis was carried out using paired *t*-test, *n* = 6 pairs. *P < 0.05, ****P < 0.0001.

The same measurements were carried out for DETA-NO–treated eyes as L-NAME–treated eyes. Results are displayed as the mean ± SD for each measurement. Results show no significant difference in the length and circumference of AAPs (*P* = 0.42 and *P* = 0.11, respectively; *n* = 6) in control eyes compared with DETA-NO–treated eyes over the short term 30-minute perfusion (Figs. 7A, 7B). The mean AAP length and circumferential length in DBG washout eyes was 21.7 ± 5.8 µm and 50.1 ± 13.1 µm, respectively, compared with 18.4 ± 8.9 µm and 42.8 ± 12.0 µm, respectively, in DETA-NO–treated eyes. In contrast, the mean DV circumferential length in DETA-NO–treated eyes seemed to be increased significantly compared with control eyes (from 21.5 ± 10.5 µm to 36.7 ± 11.0 µm; *P* = 0.03; *n* = 6) (Fig. 7C). Moreover, the number of GVs per AAP in DETA-NO–treated eyes were significantly increased compared with control (5.9 ± 1.3 vs. 9.8 ± 3.2 GVs per AAP; *P* = 0.046; *n* = 6) (Fig. 7D). Finally, the PSL in DETA-NO–treated eyes was significantly increased by nearly four -fold compared with control eyes (from 15.9 ± 10.4% to 60.7 ± 6.7%; *P* < 0.0001; *n* = 6; paired *t*-test) (Fig. 7E).

## Discussion

For more than seven decades, the cause of washout during perfusion of nonhuman eyes has been unknown. Here we present evidence that uncontrolled release of NO, associated with clamping of pressure during perfusions, is responsible for washout and morphological changes in conventional outflow tissues. Thus, we inhibited a volume-dependent increase in outflow facility (washout) in porcine eyes perfused at a constant pressure of 15 mm Hg over 3 hours with the NO synthase inhibitor, L-NAME. Coincident with functional inhibition of washout by L-NAME was absence of structural changes in outflow tissues (GV formation, DV dilation, and JCT–IW separation). Reciprocally, we mimicked washout with the short-term perfusion of the potent NO donor, DETA-NO, with a 33% increase in outflow facility over the course of the 30-minute perfusion compared with a 15% increase in DBG control eyes exhibiting washout over 3 hours. Structural changes with short-term perfusion of DETA-NO closely resembled washout changes that occur during long-term perfusions, including increased DV size and GV formation, as well as increased JCT–IW separation. This short time period was chosen to avoid too much washout in control eyes, and thus to see if acute DETA-NO exposure rapidly induces washout-like morphology. Taken together, these results support the existence of a NO-mediated pressure-sensitive homeostatic feedback loop in the conventional outflow pathway that, when short-circuited, drives washout.

Our results are consistent with previous reports of washout in the literature. Others show a washout rate of 10% to 18% increase in outflow facility from baseline after 3 hours of perfusion in whole globe porcine perfusions.^27^ Epstein et al.^51^ perfused porcine eyes at constant pressure of 15 mm Hg and DBG control eyes showed a 4% to 15% increase in outflow facility over 5 hours perfusion. Another study with pressure clamped at 15 mm Hg observed an increased in outflow facility of 19% over 9 hours of perfusion.^52^ Similarly, our study reports a washout rate of 15% increase in outflow facility in DBG control eyes over 3 hours of perfusion at 15 mm Hg. Moreover, washout was accelerated by short-term exposure to DETA-NO, resulting in a washout rate of 33%. Dismuke et al.^53^ observed a 35% increase in outflow facility after perfusion with DETA-NO (100 µM) using anterior segment perfusion organ culture with porcine eyes perfused at constant pressure of 14 mm Hg, where outflow facility increased at 10 minutes after drug delivery and reached its maximal level at 20 minutes after delivery. The fast-acting effects of DETA-NO on outflow facility observed in this study were similar to what we observed.

**Figure 8.**
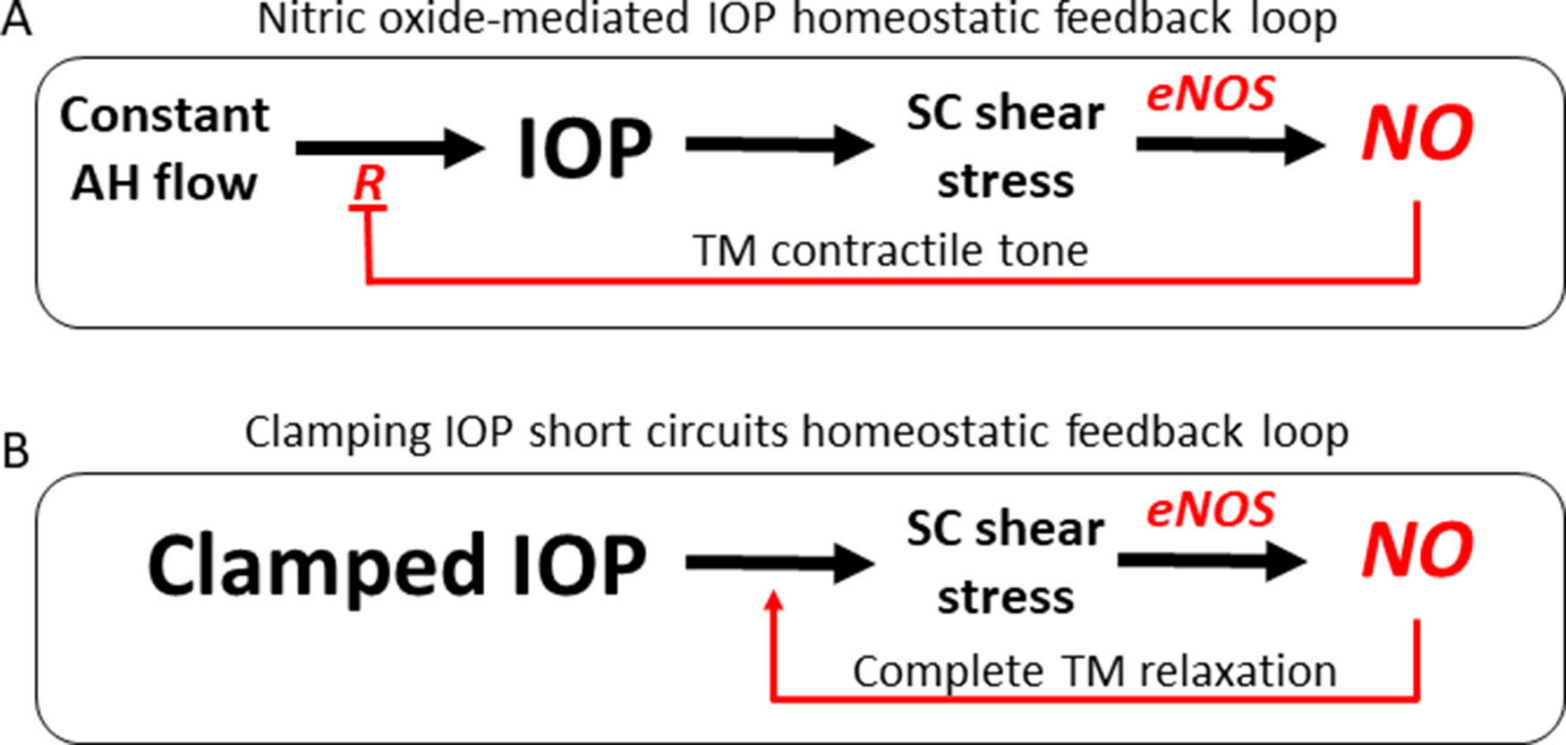
Schematic of proposed NO-mediated IOP homeostatic feedback loop and how this changes during washout. (**A**) NO-mediated IOP homeostasis feedback loop previously proposed by Reina-Torres et al.^43^ The increased IOP, caused by the increased outflow resistance, results in SC cells exhibiting shear stress owing to a mechanobiological response. This shear stress activation of eNOS results in increased NO production. This affects the TM contractile tone and results in a feedback mechanism to reduce outflow resistance (*R*) and therefore returns IOP to a more homeostatic level. This supports a model whereby NO plays an essential role as part of an endogenous homeostatic negative feedback loop regulating outflow facility, and thus IOP. (**B**) Holding the pressure constant during perfusion results in unchecked activation of eNOS and, thus, continuous NO production, owing to IOP-dependent increase in shear stress in SC (AAP) lumen. Continuous exposure of NO results in chronic relaxation and separation of the JCT region, resulting in decreased AH outflow resistance observed during washout. R, resistance to AH outflow.

As predicted by our model (Fig. 8), results showed a time-dependent increase in nitrite concentration (indicative of NO production) (Fig. 4A, Supplementary Fig. 3) in the effluent of DBG-perfused eyes exhibiting washout. During washout, the narrowing of the SC lumen is coincident with an increase in outflow facility. Because the perfusion is performed under constant pressure, the increase in facility coincides with an increase in the rate of conventional outflow. The combined increase in outflow rate through a narrowing SC lumen leads to an increased shear stress acting on the endothelial cells of SC, stimulating even more NO production and a further increase in facility. In addition to relaxation of the JCT, which occurs via retrograde diffusion, NO produced by SC cells is also carried by flow downstream to DVs, where it may contribute to DV dilation. Eventually, NO or its oxidized products, reach the ocular surface to be collected. These results are in contrast with the significantly decreased nitrite concentration in the effluent from contralateral eyes perfused with L-NAME, which did not exhibit washout.

When statistically accounting for differences between eye pairs, nitrite concentration in control eyes positively correlated with outflow facility (*P* = 0.011), which was dampened in L-NAME–treated eyes. Regardless of treatment, the potentially causal relationship between the absolute nitrite concentration in the effluent and the change in outflow facility for each eye during three one-hour intervals was visualized in Figure 4B. We hypothesized that the primary source of NO in the effluent comes from SC cells; however, the increased outflow facility during washout also results in increased shear stress in the DVs that, like SC, are shear responsive and thus can contribute to the total level of NO/nitrite in the effluent. Because of the distance and direction of the effluent flow, it is unlikely that any NO produced in the DVs is responsible for upstream effects in the JCT, where the bulk of outflow resistance is generated. Dilation of the DVs may contribute to an increase in outflow facility during washout. Therefore, our findings suggest that shear-induced NO production by SC cells is responsible for the physiological and morphological changes in the JCT (Fig. 2), whereas NO production by SC and DV endothelial cells together are responsible for DV dilation (Fig. 2). Both actions collectively likely contribute to the outflow facility increase that occurs during washout.

Endothelial NOS is critical for conventional outflow function.^45^^,^^54^ The requirement of eNOS activity was previously established using NOS3 null mice and L-NAME–treated eyes using ex vitro organ culture systems. In mice, treatment with L-NAME showed a 28% decrease in outflow facility compared with control eyes and NOS3 null mutant eyes had a 36% lower outflow facility compared with control eyes.^54^ In overexpression studies of human NOS3 in mouse eyes, outflow facility was nearly doubled and returned to wild-type levels in the presence of L-NAME.^38^^,^^44^ Finally, exogenous delivery of NO donors to porcine, monkey and human eyes during perfusions result in a 29% to 110% increase in outflow facility, consistent with our results in porcine eyes.^44^^,^^53^^,^^55^^–^^57^

The morphological effects of washout on outflow tissues can be imitated using agents that relax the TM selectively, like ROCK inhibitors. A study by Rao et al.^27^ showed increased outflow facility (40%–80%) from baseline after treatment with Y-27632 ROCK inhibitor, over the 5-hour perfusion in enucleated porcine eyes. At the 3-hour time point of this 5-hour perfusion, an 80% increase in outflow facility was observed with both 50 and 100 µM Y-27632, with increased facility reaching a plateau thereafter.^27^ This increase in outflow facility was associated with increased empty space in the JCT region and concluded that cellular relaxation and loss of cell substratum adhesions in human TM and SC cells could result in increased paracellular permeability, decreasing resistance to outflow.^27^ A study by Lu et al.^24^ also used Y-27632 to investigate effects on outflow facility, hydrodynamic patterns of outflow, and IW–JCT morphology in bovine eyes. In this study, Y-27632 increased outflow facility by 58% in treated eyes compared with control eyes. These eyes were perfused with either 0.5 mL Y-27632 or DBG.

Morphological analysis of treated eyes revealed that the IW and JCT were distended significantly compared with control eyes, with a 2.8-fold increase in PSL in treated eyes. Similar to findings with Y-27632, we reported a 3.8-fold increase in PSL in DETA-NO–treated eyes compared with control eyes, with significant separating of the IW AAP from the JCT.

In addition to JCT expansion by Y-27632, Rao et al.^27^ observed an increase in the number of GVs. Similarly, Lu et al.^24^ also reported an increase in the number of GVs in Y-27632 treated eyes compared with control eyes (2.2-fold increase). Here, we observed a 1.7-fold increase in the number of GVs in DETA-NO–treated eyes compared with control eyes. This finding is not surprising; the inhibition of the Rho/ROCK pathway can lead to rapid phosphorylation and activation of eNOS via phosphatidylinositol-3 kinase/protein kinase Akt pathway.^58^^,^^59^ Inhibition of ROCK has been shown to increase cerebral blood flow and decreased cerebral infarct size via upregulation of eNOS, potentially preventing stroke^60^; NO is a well-known mediator of vasodilation.^61^^–^^63^ As such, we observed in our study a 1.7-fold increase in DV size in DETA-NO–treated eyes compared with DBG control eyes over the 30 minutes of perfusion. This finding is consistent with functional and morphological evidence of NO regulation of DVs in the conventional outflow system.

As previously mentioned, neither human nor mouse eyes exhibit washout.^1^^,^^3^^,^^15^ Both species respond to NO treatment with increased outflow facility and decreased IOP.^38^^,^^45^^,^^64^ Moreover, IOP quickly and robustly decreases in humans after treatment with a variety of NO-donating agents.^65^^–^^69^ Although the TM and SC region are responsible for generating the majority of AH outflow resistance in the eye,^21^^,^^70^ studies have shown that completely removing the TM and IW of SC leaves approximately 25% to 50% of the resistance.^70^ It has been hypothesized that DVs play an important role in AH outflow owing to their location in series with the TM/SC.^71^ Work previously carried out in our laboratory suggests that DVs play an important role in the regulation of outflow resistance, particularly after microinvasive glaucoma surgeries, where partial removal of outflow tissue is carried out.^34^ It is, therefore, hypothesized that the effects of NO in both mice and humans is mostly caused by a relaxation of cells in the JCT, but partially caused by vasodilation of DVs by NO, a potent vasodilator.^72^ As such, the treatment of porcine eyes with DETA-NO increased DV size in this study, along with increased GV formation and separation of IW-JCT region, all of which likely play a role in decreasing AH outflow resistance.

Similar to the systemic vasculature, shear stress in SC/AAP regulates NO production.^39^^,^^73^^–^^76^ As IOP increases in the eye, the TM and the IW of SC move toward the outer wall of the SC, thereby narrowing its lumen and dramatically increasing shear stress/NO production.^44^ The IOP is hypothesized to normalize in part by an NO-mediated relaxation of underlying TM cells, an increase in the permeability of SC, and dilation of DVs.^75^^,^^77^ Importantly, all of these changes were observed in the present study during washout, when pressure was clamped (Fig. 8). Thus, the data presented here and previously by others support a model whereby NO plays an essential role as part of an endogenous homeostatic negative feedback loop regulating outflow facility and, thus, the IOP.^43^^,^^44^ A better understanding of this NO feedback loop on outflow homeostasis and, thus, the IOP can only improve strategy towards directly targeting the JCT region, the site of aberrant outflow resistance responsible for ocular hypertension in glaucoma.

## Supplementary Material

Supplement 1
