# Supplementary material for: Pressure Clamping During Ocular Perfusions Drives Nitric Oxide-Mediated Washout
Source: Invest Ophthalmol Vis Sci. 2023 Jun 26;64(7):36. doi: 10.1167/iovs.64.7.36 (PMC10297780; doi:10.1167/iovs.64.7.36)
Supplement: Supplement 1 [file iovs-64-7-36_s001.pdf]

Pressure clamping during ocular perfusions drives nitric oxide-mediated washout

Ruth A. Kelly[1], Fiona S. McDonnell[1,2], Michael L. De Ieso[1], Darryl R. Overby[3] and W. Daniel Stamer[1]

[1]Ophthalmology Department, Duke University, Durham, North Carolina, USA.
[2]Ophthalmology Department, University of Utah, Utah, USA.
[3]Department of Bioengineering, Imperial College London, London, UK.
Correspondence: W. Daniel Stamer, Duke University, Durham, NC 27707, USA;
dan.stamer@duke.edu

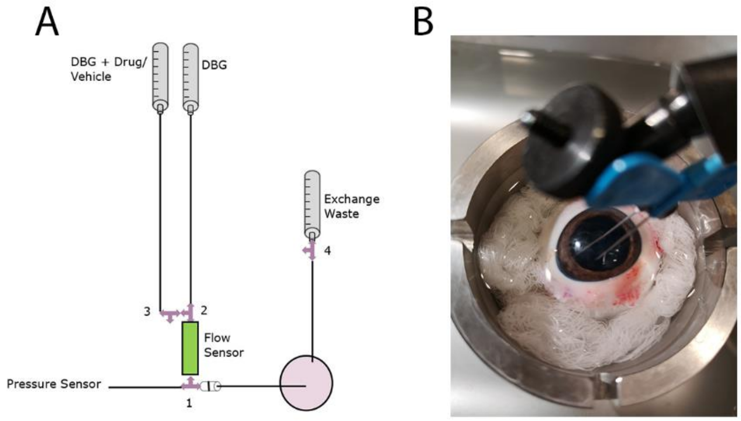

Supplemental Figure 1: Schematic of the modified version of iPerfusion system and cannulated whole porcine eye. A modified version of the iPerfusion system was used for whole porcine perfusions. (A) A schematic of one side of the system, connected to one of the paired eyes is shown. Reservoirs delivering drug/vehicle or DBG alone were clamped at a pressure of 15 mmHg. Flow readings were measured by the flow sensors and pressure readings via the pressure sensor. (B) The whole porcine eye received drug/vehicle through the posterior chamber cannulation, while the second cannulation into the anterior chamber was used for monitoring pressure, and drug/vehicle exchange.

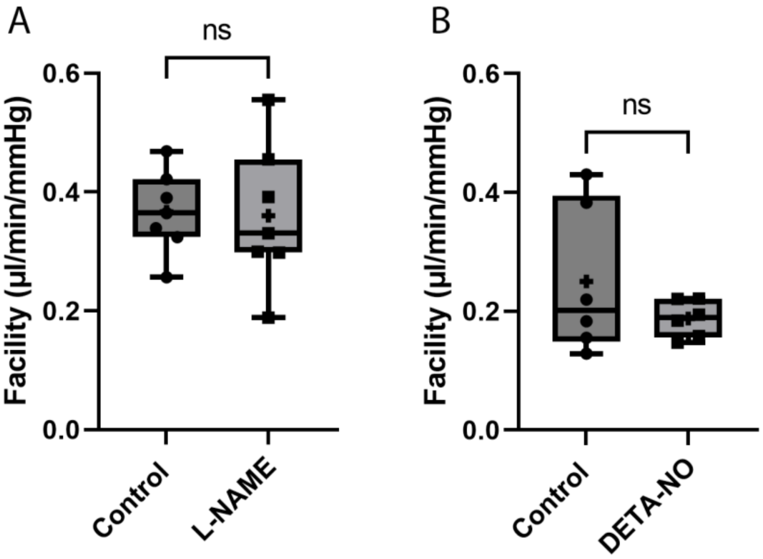

Supplemental Figure 2: Baseline facility values for both L-NAME and DETA-NO groups. (A) Mean baseline facility values for control and treated eyes were 0.37 ± 0.1 and 0.36 ± 0.1 µl/min/mmHg, respectively, in the L-NAME cohort (*p*=0.9034, t-test, n=7). (B) For comparison, mean baseline facility values for control and treated eyes were 0.25 ± 0.1 and 0.19 ± 0.03 µl/min/mmHg, respectively, in the DETA-NO cohort (*p*=0.2661, t-test, n=6) that was conducted 5 months after the L-NAME cohort. Box and whisker plots display min to max, with mean displayed as + and horizontal line in box indicates median on each graph at end of perfusion. Statistical analysis was carried out using one sample t-test.

We have found that this difference in baseline facility is not unusual; we have observed differences in baseline facility values between cohorts (±0.15 µl/min/mmHg variation) before when cohorts are carried out at separate times of the year on different groups of animals. For example, we observed mean facility values of 0.49 ± 0.2 and 0.37 ± 0.2 µl/min/mmHg in the cohort of eyes that were just run to address reviewer 2 comments for this revised manuscript (p=0.3932, paired t-test, n=4), which were similar to the first cohort (Supp. Table 1). As an aside, seasonal-dependent changes in humidity and other environmental factors has previously been linked to changes in outflow facility in mice [1].

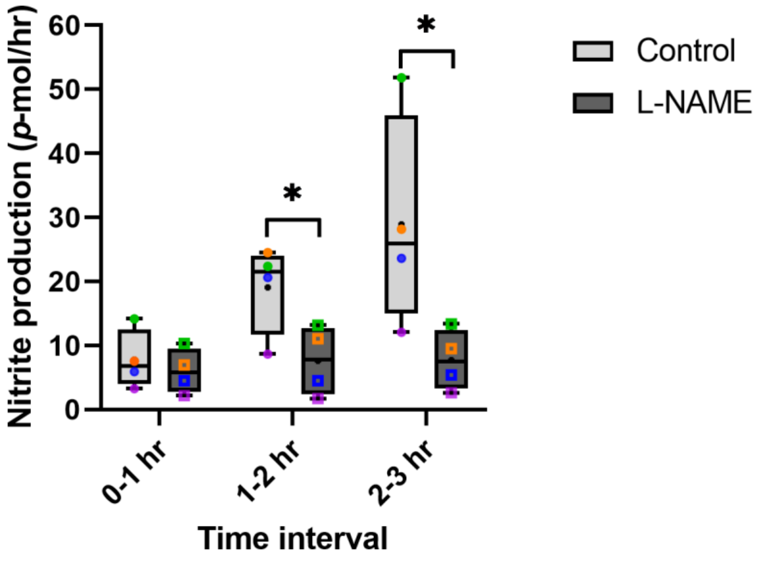

Supplemental Figure 3: Increased nitrite production rate observed over time in effluent from perfused control versus L-NAME-treated eyes. In a separate group of porcine whole globes perfused at constant pressure of 15 mmHg (n=4), effluent was collected once every hour during the perfusion and analyzed for nitrite content to estimate nitric oxide production by conventional outflow cells. In order to calculate nitrite production, the volume of effluent collected at each hourly time interval was measured. Increased nitrite production (*p*-mol/hr) was observed over time in DBG control eyes compared to L-NAME treated eyes with significance observed at both 1-2 hr (p=0.0114, paired t-test, n=4) and 2-3 hr time intervals of perfusion (p=0.0406).

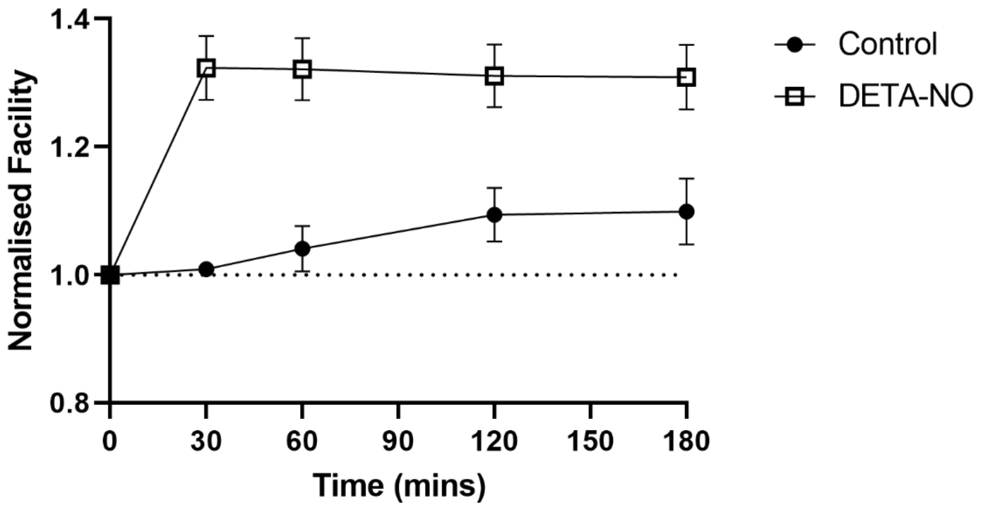

Supplemental Figure 4: Pharmacodynamics of DETA-NO during porcine perfusions. Paired eyes were perfused at constant pressure for three hours (n=3). Control eyes of pair exhibited steady washout (increase in outflow facility) rate from baseline over the course of the three hour perfusion. DETA-NO (100 nM) treated eyes however, showed an increase in outflow facility of ~30% from baseline at 30 min and this plateaued out over the 3 hr perfusion. The 30 min time point was therefore chosen for these perfusions, due to the fast-acting nature of DETA-NO. Individual data points represent mean values with ± SD error bars.

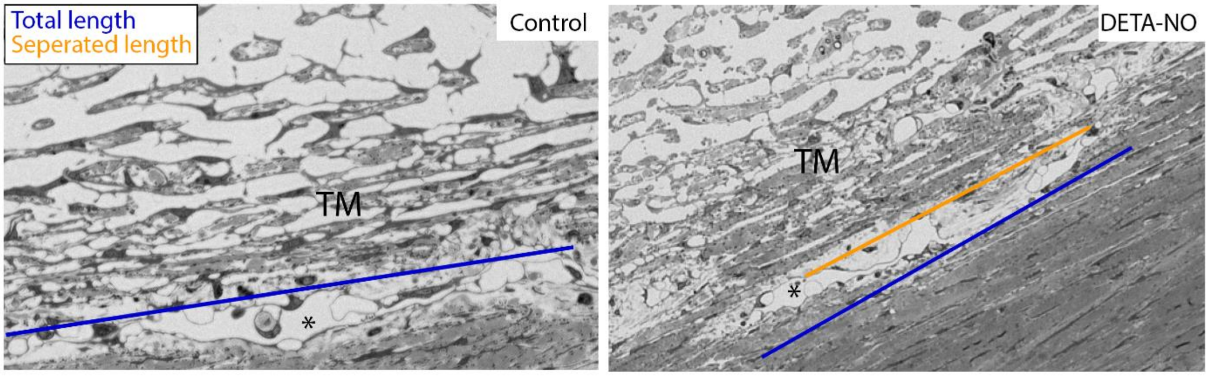

Supplemental Figure 5: Calculating percentage separation length for each AAP. Percentage separation length (PSL) was calculated to quantify the separation of the IW AAP from the JCT. The total length of the AAP (blue) and the separated length (orange) were measured using the straight line tool on ImageJ. The separated length was identified as the area of the IW of the AAP that was separated from the first JCT beam and expanded into the AAP lumen itself. PSL was calculated as a percentage of the total length of the AAP using the following equation, PSL = (separated length of AAP/total length of AAP)*100. TM=trabecular meshwork and *=angular aqueous plexus.

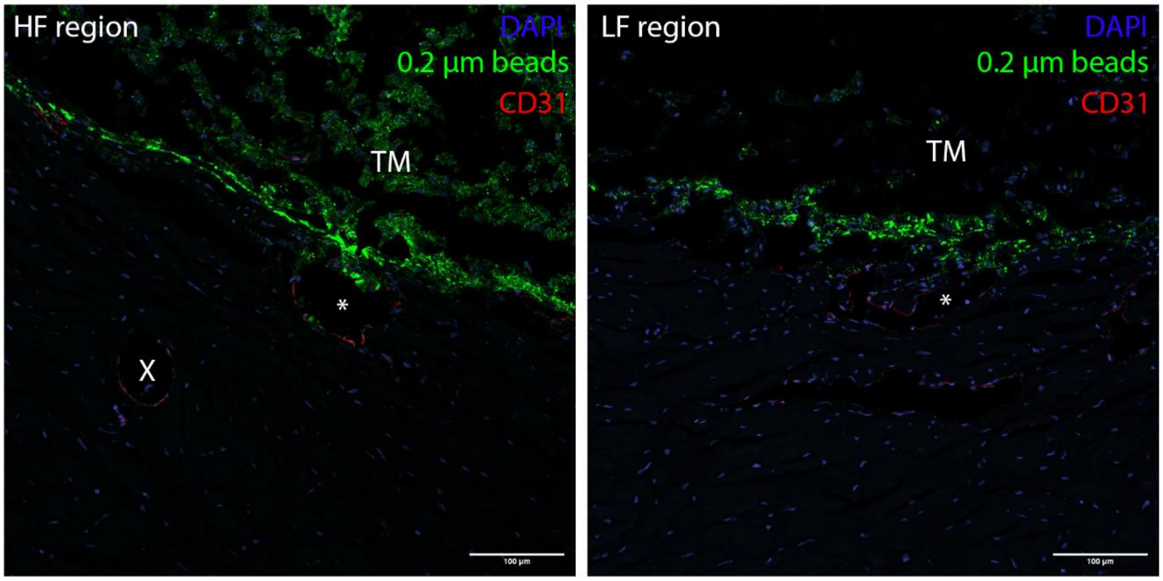

Supplemental Figure 6: Identifying conventional outflow tissues with high to medium/low amounts of tracer labeling in sagittal sections. Eyes were analyzed for morphological changes of conventional outflow tissues with medium to high amounts of tracer labeling (0.2 µm beads) in sagittal sections, indicating active high flow regions (HF, left). Low flow regions (LF, right) were not used to study morphological changes as little to no drug/vehicle would have passed through the outflow tissue. DAPI staining in blue and 0.2 µm green fluorescent labelling beads in green. TM= trabecular meshwork. *= AAP and X=distal vessel were identified using CD31 staining (red).

DETA-NO treated eyes showed greater fluorescent labeling along the IW of AAP, with more uniform labeling and effective filtration length, as previously described by Lu et al [2]. More segmental distribution of tracers and less effective filtration length was observed along the IW of AAP in both L-NAME treated eyes and 30 min DBG control eyes. Similar to findings by Lu et al [2] where they found that greater flow occurred in areas with dissociation between JCT and SC IW.

Nitrite concentration (nM)								
	**Pair 1** (Purple)		**Pair 2** (Green)		**Pair 3** (Orange)		**Pair 4** (Blue)	
**Time (Min)**	Control	L-NAME	Control	L-NAME	Control	L-NAME	Control	L-NAME
**60**	82.5	87.5	355	295	190	232.5	295	227.5
**120**	217.5	85	447.5	330	490	277.5	687.5	227.5
**180**	302.5	130	942.5	445	512.5	237.5	675	217.5
**Facility values (µl/min/mmHg)**								
**Baseline**	0.49	0.37	0.51	0.44	0.70	0.69	0.29	0.34
**60 min**	0.52	0.31	0.54	0.49	0.81	0.66	0.23	0.28
**120 min**	0.57	0.29	0.60	0.47	0.77	0.59	0.30	0.3
**180 min**	0.60	0.29	0.65	0.43	0.79	0.53	0.32	0.30

Supplemental Table 1: Nitrite concentration from total effluent collected at each hourly time point (nM) (top) and average facility values (µl/min/mmHg) (bottom) of DBG control and L-NAME treated eyes.

1. Reina-Torres, E., et al., *Reduced humidity experienced by mice in vivo coincides with reduced outflow facility measured ex vivo.* Exp Eye Res, 2019. **186**: p. 107745.
2. Lu, Z., et al., *The mechanism of increasing outflow facility by rho-kinase inhibition with Y-27632 in bovine eyes.* Exp Eye Res, 2008. **86**(2): p. 271-81.
